# MICAL1 controls cell invasive phenotype via regulating oxidative stress in breast cancer cells

**DOI:** 10.1186/s12885-016-2553-1

**Published:** 2016-07-18

**Authors:** Wenjie Deng, Yueyuan Wang, Luo Gu, Biao Duan, Jie Cui, Yujie Zhang, Yan Chen, Shixiu Sun, Jing Dong, Jun Du

**Affiliations:** Department of Physiology, Nanjing Medical University, Nanjing, 211166 China; Jiangsu Key Lab of Cancer Biomarkers, Prevention and Treatment, Collaborative Innovation Center For Cancer Personalized Medicine, Nanjing Medical University, Nanjing, 211166 China; Department of Biochemistry and Molecular Biology, Nanjing Medical University, Nanjing, 211166 China

**Keywords:** MICAL1, ROS, Invasion, Breast cancer, EGF, RAB35

## Abstract

**Background:**

Molecules Interacting with CasL (MICAL1), a multidomain flavoprotein monoxygenase, is strongly involved in the mechanisms that promote cancer cell proliferation and survival. Activation of MICAL1 causes an up-regulation of reactive oxygen species (ROS) in HeLa cells. ROS can function as a signaling molecule that modulates protein phosphorylation, leading to malignant phenotypes of cancer cells such as invasion and metastasis. Herein, we tested whether MICAL1 could control cell migration and invasion through regulating ROS in breast cancer cell lines.

**Methods:**

The effects of depletion/overexperssion of MICAL1 on cell invasion rate were measured by matrigel-based transwell assays. The contents of ROS in breast cancer cells were evaluated by CM_2_-DCFHDA staining and enhanced lucigenin chemiluminescence method. RAB35 activity was assessed by pulldown assay. The relationship of RAB35 and MICAL1 was evaluated by immunofluorescence, coimmunoprecipitation, immunoblotting and co-transfection techniques. Immunoblotting assays were also used to analyze Akt phosphorylation level.

**Results:**

In this study, we found that depletion of MICAL1 reduced cell migration and invasion as well as ROS generation. Phosphorylation of Akt was also attenuated by MICAL1 depletion. Likewise, the over-expression of MICAL1 augmented the generation of ROS, increased Akt phosphorylation, and favored invasive phenotype of breast cancer cells. Moreover, we investigated the effect of EGF signaling on MICAL1 function. We demonstrated that EGF increased RAB35 activation and activated form of RAB35 could bind to MICAL1. Silencing of RAB35 repressed ROS generation, prevented Akt phosphorylation and inhibited cell invasion in response to EGF.

**Conclusions:**

Taken together, our results provide evidence that MICAL1 plays an essential role in the activation of ROS/Akt signaling and cell invasive phenotype and identify a novel link between RAB35 and MICAL1 in regulating breast cancer cell invasion. These findings may provide a basis for designing future therapeutic strategy for blocking breast cancer metastasis.

## Background

MICAL1 is a member of molecules Interacting with CasL (MICAL) family discovered in 2002 [1]. In spite of its wide distribution in the nervous system [2], MICAL1 has been found expressed in various human normal cells as well as cancer cell lines, including melanoma and HeLa cells [3, 4]. Combined with the characteristic of anti-apoptosis, MICAL1 has been proven to be involved in cancer cell growth and survival regulation [3, 4]. MICAL1 has four conserved domains: an N-terminal flavin adenine dinucleotide (FAD) binding domain, a calponin homology (CH) domain, a Lin11, Isl-1 and Mec-3 (LIM) domain and a C-terminal coiled-coil (CC) domain, where the FAD domain is responsible for the major portion of the MICAL1’s function [5]. Studies have showed that FAD domain of MICAL1 contains flavin mono-oxygenase activity and has the ability to produce ROS [3, 6]. It has been well documented that increased oxidative stress and ROS production is crucial for breast cancer development and maintenance of its malignant state [7, 8]. Results from our studies have also shown that when breast cancer cells receive signals from their microenvironment, such as EGF, LPA and hypoxia, ROS level in cells may increase and functions as second messengers in intracellular signaling cascades which induce their migratory and invasive properties [9–11]. However, whether MICAL1 could influence cell metastatic property by regulating ROS level in breast cancer cells remains to be determined.

It is now known that MICAL1 displays an auto-inhibitory mechanism to control its biomolecular function. Normally, the MICAL1 CC domain binds to its LIM domain to mediate the auto-inhibition. Removal of the CC domain from MICAL1 or affect the binding of CC domain to its LIM domain may cause the activation of its mono-oxygenase domain, leading to ROS production and F-actin assembly alteration. Actually, MICAL1 is a highly regulated protein, the auto-inhibition state could be relieved by the interaction occur within CC domain and other proteins such as RAB1 and Plexin under various cellular conditions [12, 13]. RABs are the largest family of small GTPases and involved in the control of intracellular membrane trafficking and cell motility through interaction with specific effector molecules [14, 15]. By a yeast two-hybrid assay, previous study has systematically screened the ability of MICAL1 binding for all the members of Rab family and found that GTP-bound RAB35 was one of the few members which interacted strongly with MICAL1 [16].

Like all GTPases, RAB35 activity is under tight control, which is mediated by guanine nucleotide exchange factors (GEFs) and GTPase activating proteins (GAPs) that catalyze GTP exchange and hydrolysis, respectively [14]. Notably, RAB35 has ample opportunities to influence diverse cell signaling, resulting in functional promiscuity on tumor initiation and progression, and the activity of RAB35 in tumor is of tremendous research interest. It has been shown that RAB35 functions as a tumor suppressor and attenuated signaling downstream of Arf6 [17]. However, Studies on *Drosophila* cultured cells have led to the suggestion that RAB35 may promote the assembly of actin filaments during bristle development and increase filopodia formation [18]. Similarly, there are also report that RAB35 is over-expressed in ovarian cancer [19]. Recent studies including the results from our laboratory also showed that RAB35 activation could be act as a positive regulator of cell shape, phagocytosis as well as migration in various types of cells [20–22]. Several studies have highlighted a link between RAB35 and MICAL-l1, a similar protein to MICAL1, which revealed that RAB35 could use MICAL-l1 as its membrane hub effector [23, 24]. Although RAB35 could recruit different effectors to perform specific biological process, it remains unclear whether and if so, the biological relevance of RAB35 binding to MICAL1 in breast cancer cells. In this study, we examined whether knockdown or overexpression of MICAL1 could influence ROS generation and cell migration firstly, and then explored the mechanism underlying MICAL1 action by examining the effect of RAB35 blockage/activation on those process.

## Methods

### Cell and plasmids

Human breast cancer cell lines MDA-MB-231, MCF-7, T47D, BT474 and MDA-MB-468 were obtained from the Cell Biology Institute of Chinese Academy of Sciences (Shanghai, China). Cells were cultured in Dulbecco’s modified Eagle’s medium (DMEM, high glucose) (Hyclone, Thermo Scientific, Waltham, MA, USA) supplemented with 10 % (v/v) fetal bovine serum (FBS) (Hyclone) and antibiotics (100 U/mL streptomycin and 100 μg/mL penicillin) (Invitrogen, Carlsbad, USA) in a humidified incubator at 37 °C with 5 % CO_2_. Cells were grown on coverslips for fluorescence staining and on plastic dishes for protein extraction. Cells were made quiescent by serum starvation overnight followed by EGF (R&D Systems, Minneapolis, MN, USA) treatment.

The RAB35-Q67L (constitutively active, CA), RAB35-S22N (dominant negative, DN) and wild-type RAB35 (WT) plasmids were kindly provided by Dr. Matthew P. Scott (Department of Developmental Biology, Stanford University, USA). The PCR products were cloned into the pEGFP-N1 vector (Clontech, Palo Alto, CA, USA). Human MICAL1 cDNA clone was purchased from Youbio (Hunan, China). The full-length MICAL1 DNA was amplified from pOTB7-MICAL1 plasmid using the following primer set, sense: 5′-CCCAAGCTTGCCACCATGGCTTCACCTACCTCCA-3′, antisence: 5′-CCAACTCGAGGCCCTGGGCCCCTGTCCCCAAGGCCA-3′. In these primers, Hind III and Xho I restriction site sequences have been underlined. The polymerase chain reaction (PCR) products were cloned into the pCMV-C-HA vector (Beyotime, Nantong, China). Truncated MICAL1 lacking CC domain (residues 1–799) and truncated MICAL1 containing CC domain (residues 800-1068) were also created as previously described [3]. The cells were seeded in 6-well plates, cultured to 80 ~ 90 % confluence, and then transiently transfected with those plasmids by using FuGENE HD Transfection Reagent (Promega Corporation, Madison, WI, USA) according to the manufacturer’s instructions.

### siRNA knockdown studies

The sequences of small interfering RNA (siRNA) for MICAL1 were as follows: #1, 5′-GUCUCUGCCUUUGACUUCATT-3′, #2, 5′-CUGCAGAACAUUGUGUACUTT-3′, and #3, 5′-CUCGGUGCUAAGAAGUUCUTT-3′; siRNA for RAB35 was: 5′-GCAGCAACAACAGAACGAUTT-3′ and the sequence of control siRNA was 5′-UUCUCCGAACGUGUCACGUTT-3′ (GenePharma, Shanghai, China). Cells were transfected with siRNA by Lipofectamine 2000 according to the manufacturer’s instruction.

### Migration and invasion assays

For wound healing assay, breast cancer cells were seeded in a 96-well plate. Approximately 24 h later, when cells were 95 ~ 100 % confluent, cells were incubated overnight in DMEM and wounding was performed by scraping through the cell monolayer with a 10 μl pipette tip. Medium and nonadherent cells were removed, and cells were washed twice with PBS, and new medium with or without EGF was added. Cells were permitted to migrate into the area of clearing for 18 h. Wound closure was monitored by visual examination under microscope (Carl Zeiss Meditec).

For transwell migration assay, breast cancer cells in exponential growth were harvested, washed, and suspended in DMEM without FBS. Cells (2 × 10^5^/200 μl) were seeded into polycarbonate membrane inserts (8 μm pore size) in 24-transwell cell culture dishes. Cells were allowed to attach to the membrane for 30 min. The lower chamber was filled with 600 μl DMEM with EGF or with 10 % FBS. Cells were permitted to migrate for 12 h. After the incubation, stationary cells were removed from the upper surface of the membranes. The cells that had migrated to the lower surface were fixed and stained with 0.1 % crystal violet. Cell invasion was analyzed using the same protocol as for cell transwell migration, but with the use of matrigel (BD Bioscience) pre-coated cell culture inserts. Cells were permitted to invade for 24 h.

### Coimmunoprecipitation and immunoblotting assays

Coimmunoprecipitation assays were performed as previously described. Briefly, cell lysates were incubated with antibody at 4 °C overnight. Antibody-bound complexes were precipitated with protein A + G agarose beads (Beyotime) and eluted by rinsing buffer, then the agarose-associated protein complexes were dissolved in SDS loading buffer and analyzed by immunoblotting assays.

Sample protein extraction and concentration determination of whole cells were performed as previously described [9]. Briefly, equal amounts of protein were run on SDS polyacrylamide gels and transferred to nitrocellulose membrane. The resulting blots were blocked with 5 % non-fat dry milk and probed with antibodies. The following antibodies were used: GAPDH (KangChen), MICAL1 (proteintech) (Santa Cruz Biotechnology), RAB35 (BD Biosciences) (ABclonal Technology), Akt, P-Akt, HA and GFP antibodies (Cell Signaling). Protein bands were detected by incubating with HRP-conjugated secondary antibodies (Santa Cruz Biotechnology) and visualized with ECL reagent (Millipore).

### Pulldown assays

RAB35 activity was measured by pulldown assays as described previously [22]. In brief, the GST fusion RBD35 was purified from BL21 bacteria and incubated with cell lysates. Then the complexs were incubated with MagneGST Glutathione Particles (Promega) for 30 min on a rotating wheel at 4 °C. After washed with washing buffer and collected by magnet in a magnetic stand (Promega), the beads were solubilized in 2 × SDS loading buffer, and then subjected to immunoblotting assays with antibody against RAB35.

### Immunofluorescence and immunohistochemistry assays

Cells used for immunostaining were fixed in ice-cold methanol for 20 min, permeabilized in 0.1 % Triton X-100 and blocked in PBS containing 1 % BSA for 1 h at room temperature. The cells were incubated with primary antibody overnight at 4 °C followed by incubation with FITC or rhodamine conjugated secondary antibody for 1 h at room temperature within a moist chamber. After wash with PBS, the samples were mounted with DAPI Fluoromount G (Southern Biotech). Images were acquired using an Olympus BX51 microscope coupled with an Olympus DP70 digital camera.

### Measurement of ROS

For intracellular H_2_O_2_ staining, 1 × 10^5^ cells were seeded on a coverslip placed in a 6-well plate and incubated overnight. After treated with appropriate inhibitors and stimuli as detailed elsewhere in the text, the cells were stained with 5 μM 2′,7′-dichlorofluorescein diacetate (CM_2_-DCFHDA) (Invitrogen) for 15 min at 37 °C. After wash with PBS, the cover slips were mounted on glass slides. Images were collected using an Olympus BX51 microscope coupled with an Olympus DP70 digital camera.

The level of superoxide anions in the cells was measured using the enhanced lucigenin chemiluminescence method. The homogenate supernatant of total cellular protein was diluted in modified HEPES buffer. The reaction started by addition of 5 μM dark-adapted lucigenin (Sigma). Light emission was measured for 10 times in 10 min with a luminometer (20/20n, Turner), and average values were calculated and expressed as mean light unit (MLU) per min per milligram of protein, which represented the level of superoxide anions.

### Statistical analysis

Statistical analysis was performed using the SPSS statistical software program (Version 19.0; SPSS, Chicago, IL, USA). Error bars represent standard error of mean S.E.M, the significance of difference in two groups was analyzed by Student’s *t* test. *P* < 0.05 represents statistical significance and *P* < 0.01 represents sufficiently statistical significance (two tailed).

## Results

### MICAL1 regulates breast cancer cell migration and invasion

**Fig. 1:**
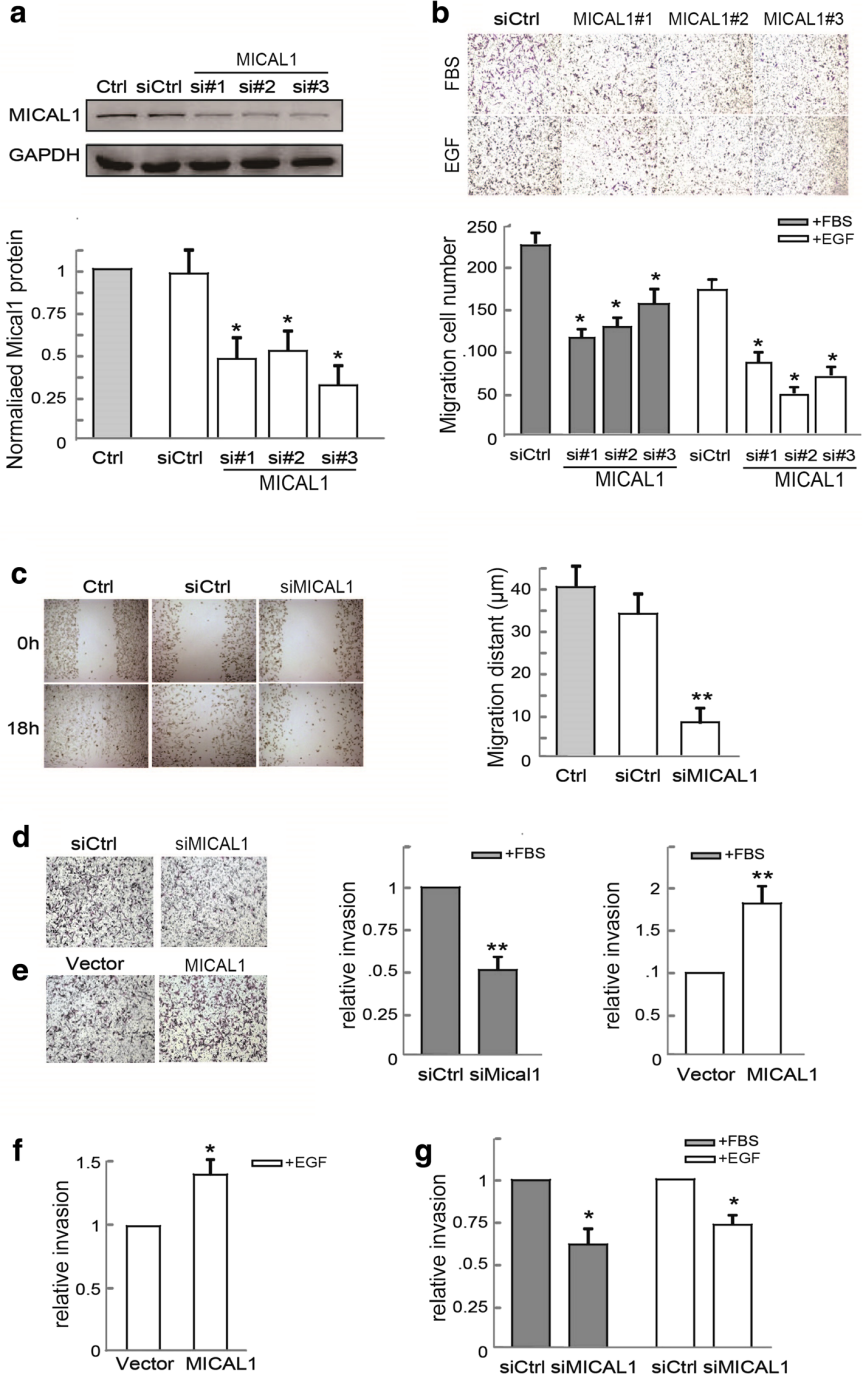
MICAL1 regulates breast cancer migration and invasion in vitro. **a** MDA-MB-231 cells were transfected with negative control siRNA or siRNA specifically targeting MICAL1 (siMICAL1). 48 h later, total protein extracts from cells were analyzed by immunoblotting analysis for MICAL1 expression. *: *P* < 0.05 in the cells transfected with or without siRNA targeting MICAL1. (**b**&**c**) Representative transwell migration (**b**) and wound healing assay (**c**) images of control and MICAL1 silencing MDA-MB-231 cells. Quantification of migration rates was analyzed respectively. **d** MDA-MB-231 cells transfected with control siRNA or siMICAL1, and the quantification of cell invasion rate was performed. (**e**&**f**) MDA-MB-231 (**e**) and MCF-7 cells (**f**) were transfected with MICAL1 or empty vector, and the quantification of cell invasion rate was performed. *:*P* < 0.05, **:*P* < 0.01 in the cells transfected with HA–MICAL1 relative to cells transfected with the corresponding vector. **g** MCF-7 cells transfected with control siRNA or siMICAL1, and the quantification of cell invasion rate was performed. *: *P* < 0.05 in the siMICAL1 cells relative to siRNA control cells

To examine the function of MICAL1 in breast cancer progression, we silenced MICAL1 expression in human breast cancer MDA-MB-231 cells with siRNA for MICAL1. The cells were lysed and the knockdown efficiency was determined by immunoblotting assays (Fig. 1a). Enhanced motility of breast cancer cells is a critical step in promoting tumor metastasis, but roles of MICAL1 in breast cancer motility remain to be determined. We first explored the effects of MICAL1 silencing on breast cancer cell migration in vitro. By wound healing assay and transwell migration assay, we found that siMICAL1-transfected MDA-MB-231 cells exhibited decreased migratory potential than the control cells (Fig. 1b&c). To confirm the role of MICAL1 in regulating breast cancer cell motility, we also performed the same transfection (siMICAL1 #3) in MDA-MB-231 and MCF-7 cells, and found that silencing MICAL1 also inhibited cell invasion in those cells (Fig. 1d&g). In contrast, increased invasive potential was observed in both cells overexpressed MICAL1 (Fig. 1e&f). These results indicate that MICAL1 plays a positive role in regulating breast cancer cell migratory and invasive potential.

### MICAL1 forms complexes with active form of RAB35

**Fig. 2:**
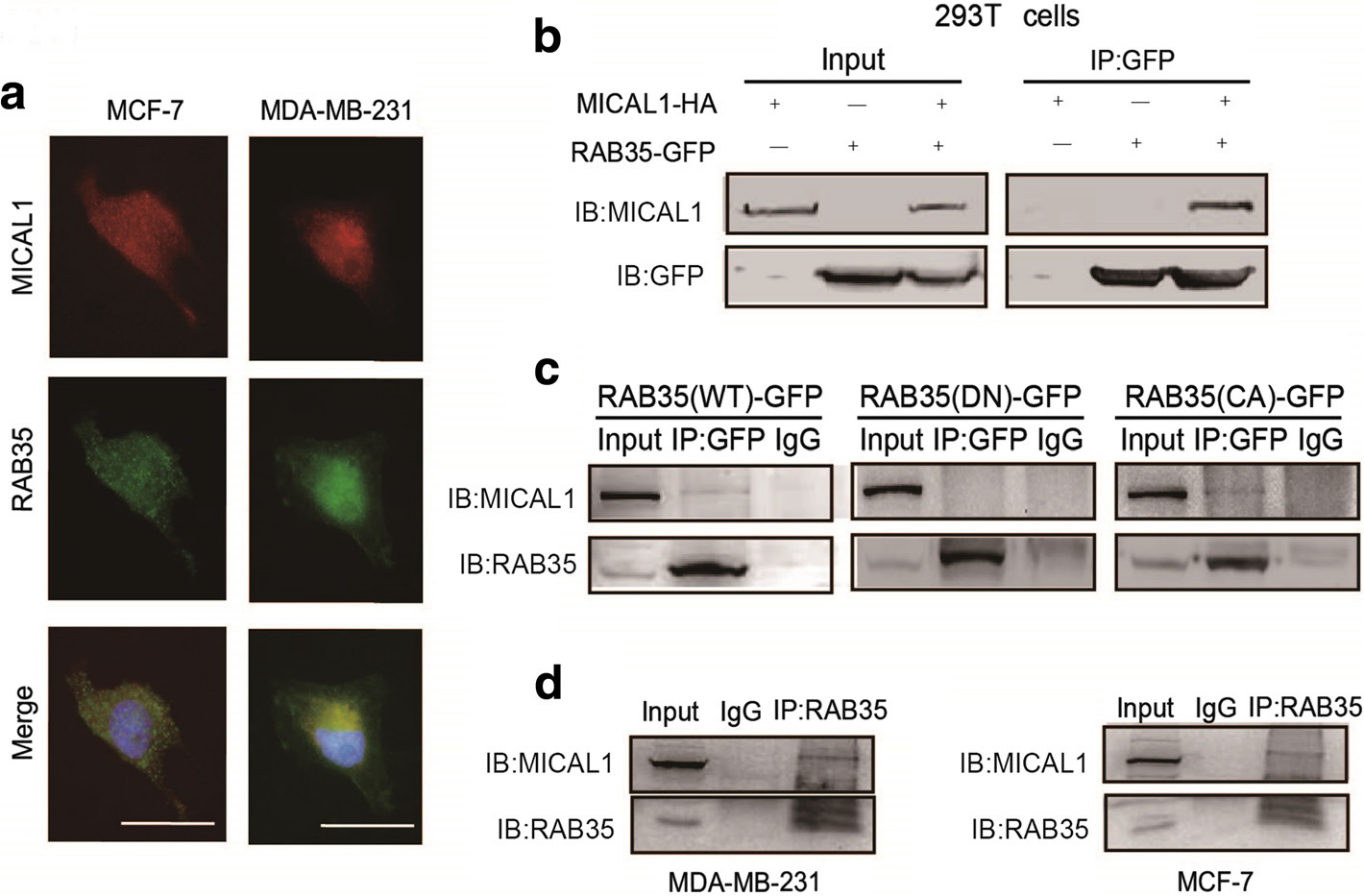
Active form of RAB35 binds to MICAL1. **a** Representative micrographs of MDA-MB-231 and MCF-7 cells stained for RAB35 (green) and MICAL1 expression (red) by immunofluorescence assay. Scale bar, 10 μm. **b** Coimmunoprecipitation experiments were performed with HEK293T cells cotransfected with HA-tagged MICAL1 and GFP-tagged RAB35. **c** MCF-7 cells were transfected with GFP-tagged RAB35 (WT), RAB35 (DN) or RAB35 (CA), and then immunoprecipitated with anti-GFP antibody, followed by immunoblotting analysis for RAB35 and MICAL1. **d** Binding of endogenous RAB35 to MICAL1 was detected in MDA-MB-231 cells and MCF-7 cells by coimmunoprecipitation experiments. *n* = 3 for all experiments

A number of coregulators, such as RAB1, is reported dynamically bind to MICAL1 and modulate its activity. We hypothesized that during migration, MICAL1 activity might be induced through a similar mechanism. By a yeast two-hybrid assay, GTP-bound RAB35 was identified could interacted strongly with MICAL1 [16]. In this study, immunoflurescence analysis showed that MICAL1 was partially colocalized with RAB35 in both MDA-MB-231 and MCF-7 cells (Fig. 2a). Furthermore, coimmunoprecipitation experiments were performed to determine whether RAB35 binds to MICAL1 in cells. GFP-RAB35 and/or HA-MICAL1 vector was cotransfected in HEK293T cells, and the protein complexes were immunoprecipitated by anti-GFP antibody. We noticed that a significant amount of MICAL1 was pulled down in the GFP-RAB35-expressing cells but not in the control cells, indicating that MICAL1 binds to RAB35 in HEK293T cells (Fig. 2b). The interaction between MICAL1 and RAB35 was also confirmed in MCF-7 cells, which showed that interaction between endogenous MICAL1 and RAB35 (WT) as well as active form of RAB35 (CA), but not inactive form of RAB35 (DN) (Fig. 2c). We also showed interaction between endogenous RAB35 and MICAL1 in coimmunoprecipitation assays in both MDA-MB-231 and MCF-7 cells (Fig. 2d).

### Activation of RAB35 is necessary for EGF-induced invasion

**Fig. 3:**
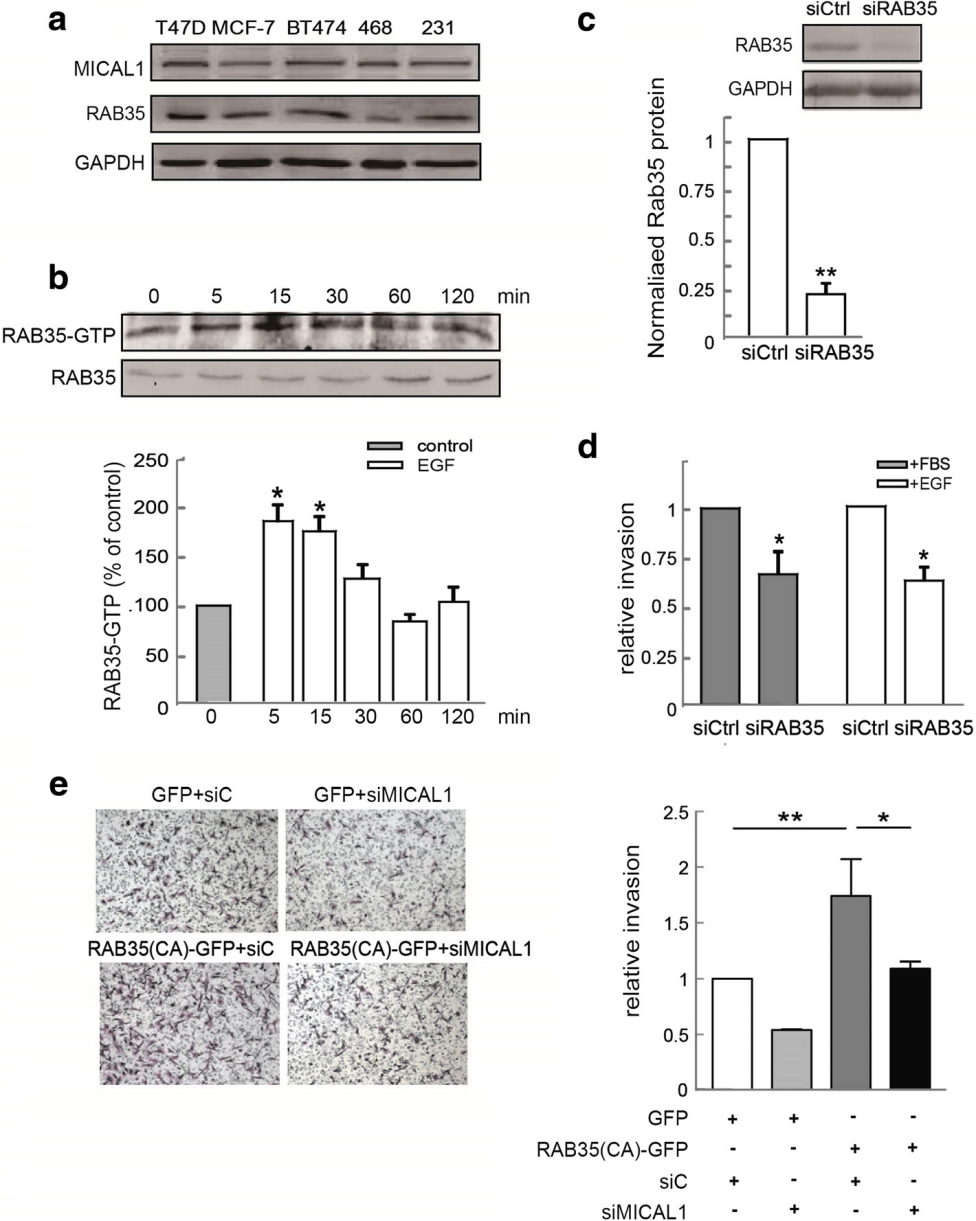
Effect of RAB35 on breast cancer cell invasion. **a** RAB35 and MICAL1 expressions were examined by immunoblotting in several types of malignant breast cancer cell line. **b** MDA-MB-231 cells were incubated with EGF (10 ng/mL) for indicated times, and analyzed for RAB35 activity by pulldown assays. *P < 0.05 in the cultures with EGF relative to the cultures without EGF. **c** MCF-7 cells were transfected with negative control siRNA or siRAB35. 48 h later, total protein extracts from cells were analyzed by immunoblotting analysis for RAB35 expression. Western blot bands corresponding to RAB35 were quantified and normalized against GAPDH level. **: *P* < 0.01 in the siRAB35 cells relative to control siRNA cells. **d** MCF-7 cells transfected with control siRNA or siRAB35, and the quantification of cell invasion rate was performed. *: *P* < 0.05 in the siMICAL1 cells relative to control siRNA cells. **e** Invasion assays results showed that knockdown of MICAL1 delayed cell invasion in RAB35 (CA)-expressing MDA-MB-231 cells. *:*P* < 0.05. **:*P* < 0.01

We screened the protein levels of MICAL1 and RAB35 in five breast cancer cell lines and found that those two proteins were abundantly expressed (Fig. 3a). RAB35 is well characterized in the Wnt5a pathway, which was identified as a potent modulator of MCF-7 breast cancer cell migration [22]. Firstly, we examined whether RAB35 could also be activated by EGF in MCF-7 cells. Immunoblotting assays showed weak but detectable steady state expression of activated RAB35, which was clearly augmentated after 5-15 min of EGF treatment (Fig. 3b). Next, to verify that RAB35 could impact cell motility, we transfected cells with siRNA against RAB35 (Fig. 3c), and examined its effect on MCF-7 cell invasion. As shown in Fig. 3d, following EGF or FBS stimulation, numbers of invasive cells were decreased significantly in the group transfected with siRAB35, compared to the cells transfected with sictrl. Invasion assays results also showed that knockdown of MICAL1 delayed the invasive ability of RAB35 (CA)-expressing MDA-MB-231 cells (Fig. 3e). Taken together, these experiments demonstrated that RAB35 was required for EGF-induced invasion in breast cancer cells.

### RAB35 and MICAL1 mediate EGF-induced ROS generation

**Fig. 4:**
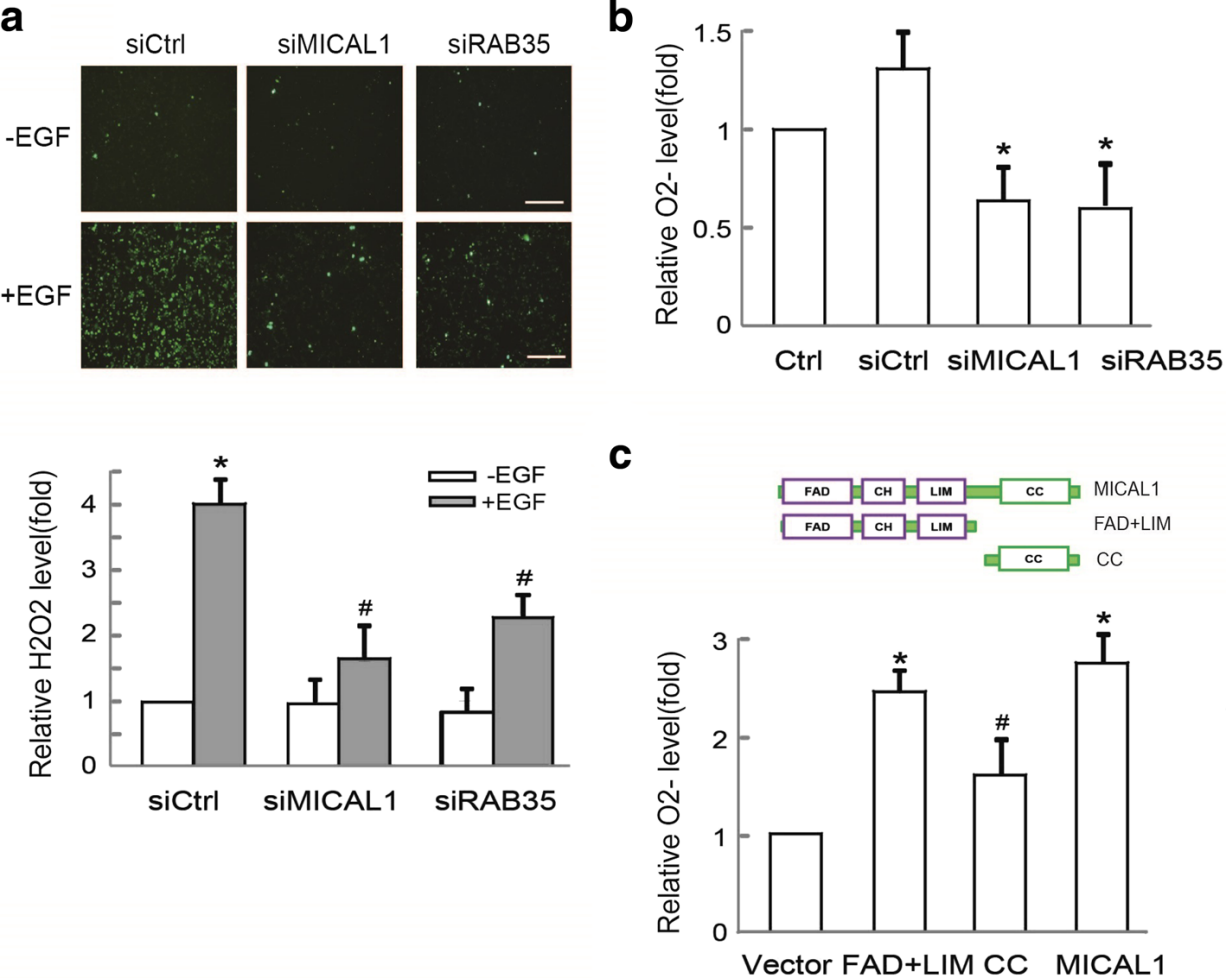
RAB35/MICAL1 regulates ROS generation. **a** Effects of RAB35 and MICAL1 on H_2_O_2_ generation. MCF-7 cells transfected with siRAB35 or siMICAL1 were in serum-free media overnight. Representative micrographs of those cells incubated with EGF (10 ng/mL for 30 min) and stained with CM_2_-DCFHDA. *: *P* < 0.05 in the cultures with EGF relative to the cultures without EGF. ^#^:*P* < 0.05 in the cultures with EGF plus siRAB35 or siMICAL1 relative to the cultures with EGF. Scale bar, 100 μm. **b** Effects of RAB35 and MICAL1 on O_2_^−^ generation in MCF-7 cells. *: *P* < 0.05 in the cultures transfected with siRAB35 or siMICAL1 relative to the cultures with control siRNA. **c** Quantification of O_2_^−^ levels in the cells transfected with HA–MICAL1, HA tagged MICAL1 FAD-LIM domain and CC domain. *: *P* < 0.05 in the cells transfected with HA–MICAL1 or HA–FAD-LIM relative to cells transfected with vector. ^#^: *P* < 0.05 in the cells transfected with CC domain relative to cells transfected with HA–MICAL1 or HA–FAD-LIM domain

MICAL1 is well characterized in the ROS generation, which has been associated with cancer cell invasion. To verify RAB35 and MICAL1 could impact EGF-mediated ROS, we transfected cells with siRAB35 or siMICAL1, and then examined its effect on ROS generation. As shown in Fig. 4a, weak but detectable steady state production of ROS was showed in control cells. After 30 min of EGF treatment, ROS level was clearly increased in the control cells transfected with the empty vector, but it was down-regulated in the cells transfected with siRAB35 or siMICAL1. Meanwhile, ROS generation in serum-cultured MCF-7 cells is also inhibited by either siRAB35 or siMICAL1 transfection (Fig. 4b). It is worth noting that cells expressing full-length MICAL1 or FAD-LIM domain of MICAL1 induced high levels of ROS in transfected cells. However, upon transfection of the CC domain, the levels of ROS were only slightly higher than the baseline levels (Fig. 4c). Taken together, these experiments demonstrated that both RAB35 and MICAL1 were required for ROS generation in breast cancer cells.

### ROS mediates RAB35/MICAL1 signals and regulates cell invasion via phosphorylated Akt

**Fig. 5:**
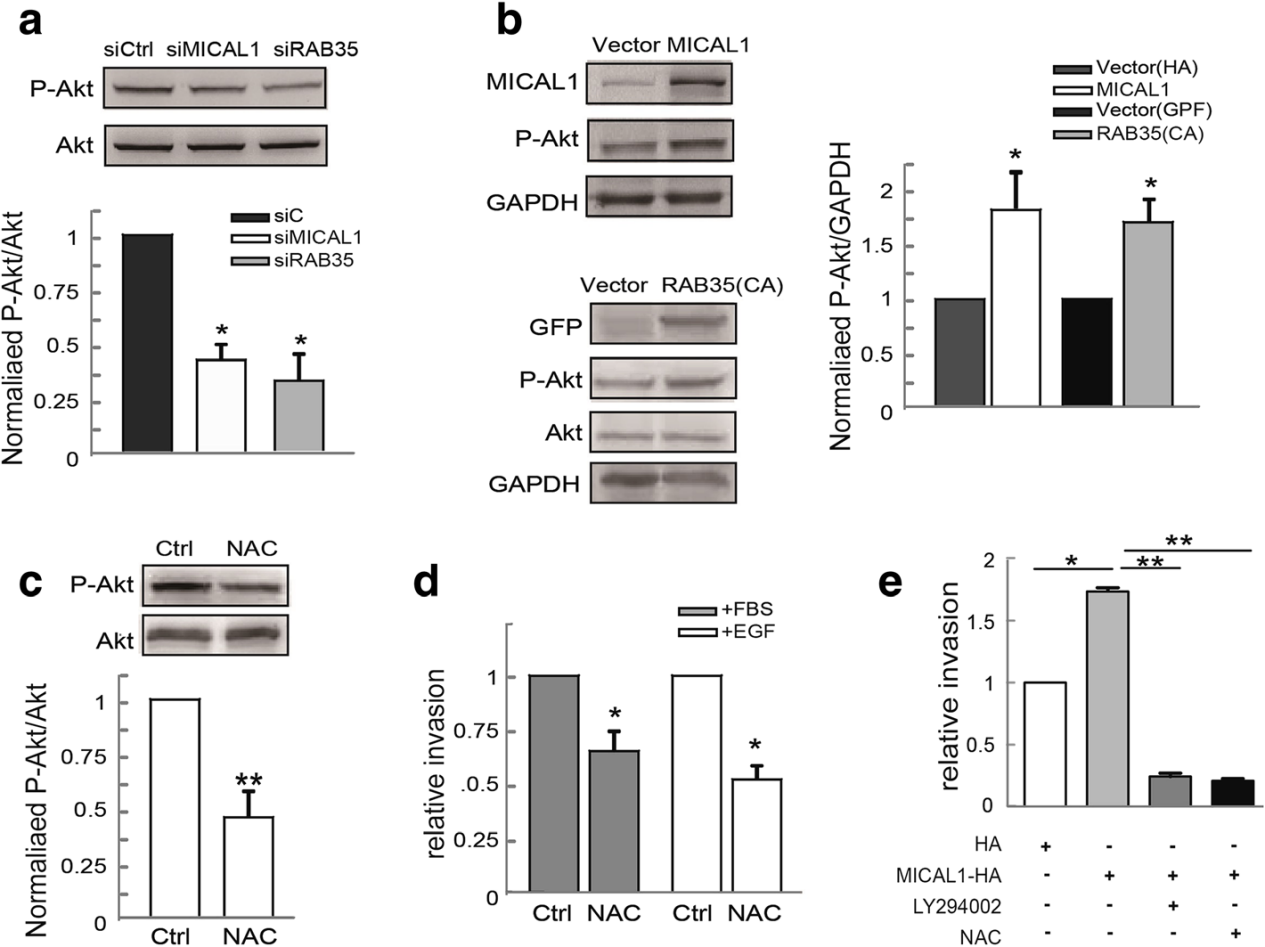
Effects of RAB35 and MICAL1 on ROS-modulated Akt activity. **a** MCF-7 cells transfected with siRAB35 or siMICAL1, and protein levels of P-Akt and Akt were examined. Western blot bands corresponding to P-Akt were quantified and normalized against Akt levels. *: *P* < 0.05 in the cultures transfected with siRAB35 or siMICAL1 relative to the cultures with control siRNA. **b** MCF-7 cells were transfected with MICAL1 or RAB35 (CA) plasmids, and the total cellular proteins were extracted and analyzed for expressions of P-Akt and Akt by immunoblotting assays. *:*P* < 0.05 in the cells transfected with HA–MICAL1 or GFP–RAB35 (CA) relative to cells transfected with the corresponding vector. **c** MCF-7 cells were treated with 2 mM NAC for 1 h, and then the total protein extracts from cells were analyzed by immunoblotting assays for P-Akt and Akt expression. **d** Effect of NAC on cell invasion. After cultured in serum-free medium overnight, MCF-7 cells were pretreated with 2 mM NAC for 1 h, and then were stimulated with EGF (10 ng/mL) or 10 % FBS for 24 h. Quantifications of cells on the lower surface of the membrane were performed and shown. *: *P* < 0.05, **: *P* < 0.01 in the cultures with NAC relative to the cultures without NAC. **e** Invasion assays results showed that LY294002 and NAC delayed cell invasion in MICAL1-expressing MCF-7 cells. *:*P* < 0.05. **:*P* < 0.01

PI3K/Akt plays a key role in migratory potential regulation. To probe the involvement of PI3K/Akt activation in RAB35/MICAL1-induced cell motility, we transfected MCF-7 cells with siRAB35 or siMICAL1 and P-Akt expression was detected by immunoblotting assays. Our observations have yielded evidence that the level of P-Akt was markedly blocked after the silencing of MICAL1. Consistently, P-Akt was higher when MICAL1 or RAB35-Q67L overexpressed in MCF-7 cells (Fig. 5a&b). Together, these results indicated that RAB35 and MICAL1 could affect the level of P-Akt. Moreover, pre-treatment with ROS inhibitor NAC inhibited P-Akt level (Fig. 5c) as well as cell invasion (Fig. 5d). NAC and LY294002 pretreatment also delayed the increased invasion activity induced by overexpression of MICAL1 (Fig. 5e). Collectively, these data indicate that the activation of RAB35/MICAL1 may facilitate ROS generation, which leading to PI3K/Akt signaling activation and breast cancer cell invasion.

## Discussion

While there is only one gene encoding MICAL in *Drosophila*, vertebrates contain three genes encoding MICAL isoforms indicated as MICAL1, MICAL2 and MICAL3. Furthermore, MICAL-like forms, which were absent of FAD domain, also have been identified exist in vertebrates. In *Drosophila*, MICAL selectively oxidizes Met 44 residue within the D-loop of actin, thereby destabilizing F-actin and inhibiting local assembly [25]. MICAL1 has the most closely related domain architecture to *Drosophila* MICAL [3], however, to date, only a few reports have been published to describe the functions of MICAL1 during cancer progression. Previous studies have shown that aberrant activation MICAL1 is a negative regulator of apoptosis and contributes to malignant progression of melanoma [4]. In the present study, we demonstrate that knockdown of MICAL1 has favorable effect on preventing cell migration and invasion. We also show that ROS acts as downstream of MICAL1 to regulate cell invasion. Moreover, we determine a novel link between RAB35 and MICAL1 in regulating EGF-induced breast cancer cell invasion. Taken together, we demonstrate for the first time that MICAL1 may play a potential role in breast cancer cell motility and shed light on new therapeutic target against breast cancer invasion and metastasis.

Enhanced motility of cancer cells is a critical ability in promoting tumor metastasis and mortality of patients. Here, we delineated the role of MICAL1 in regulating breast cancer cell motility. Our results showed that the migratory and invasive ability of breast cancer cells induced by EGF or FBS stimulation decreased significantly after MICAL1 silencing in vitro. Consistently, MICAL1 overexpression in the cancer cells accelerated their motility behavior. Recent study showed that MICAL2-positive cells peculiarly localized at the primary human gastric cancer invasive front and MICAL2 knock-down in cancer cells resulted in mesenchymal to epithelial transition [26]. Similar with those results, in the present study, we uncovered an essential role of MICAL1 in promoting migration and invasion of breast cancer cells. Given our observation that silencing MICAL1 specifically inhibited EGF induced cell invasion, it will be interesting to elucidate the exact mechanisms by which EGF regulate MICAL1’s function.

ROS are highly reactive molecules generated by incomplete reduction of oxygen, including superoxide, hydrogen peroxide, and hydroxyl radical et al. Of note, increased oxidative stress and ROS production was present in many human metastatic tumors, and the roles of ROS in triggering signaling pathways for cell migration and invasion have been well established [27, 28]. Here, we demonstrated that the increased level of ROS production after EGF stimulation was markedly blocked by silencing of MICAL1. Moreover, cells only expressing CC domain from MICAL1 displayed lower levels of ROS when compared with cells overexpressed full-length of MICAL1. Our finding is consistent with a previous report that HeLa cells transfected with the FAD domain from MICAL1 augmented ROS levels [3]. Consistently, the levels of ROS were significantly attenuated upon transfection of the enzymatically impaired FAD domain mutant [3]. Therefore, it is proposed that during EGF stimulation, MICAL1, especially for its FAD domain, facilitates the production of ROS, helping to promote the migratory and invasive ability of breast cancer cells.

As due to their very nature, ROS cannot impart cell migratory functions directly. Activation of PI3K/Akt by ROS was shown be an important mechanism to mediate breast cancer cell migration by LPA [9]. In keeping with this idea, MICAL1-induced breast cancer cell invasion might be PI3K/Akt dependent. Our observations have yielded evidence that the increase of P-Akt was markedly blocked after the silencing of MICAL1. Consistently, P-Akt was higher in MICAL1 overexpressed breast cancer cells. It is worth noting that the effect of ROS on lung cancer cell migratory functions is dependent on Akt activity [29]. Here, we also found that P-Akt level as well as cell invasion was blocked by application of ROS scavenger NAC. Therefore, Akt is more likely the target of ROS downstream of MICAL1 to regulate breast cancer cell motility.

RAB35 may functions downstream of growth factor receptors and associates with PI3K. Further, the expression of GTP-bound RAB35 is necessary and sufficient for PI3K/Akt signaling activation and apoptosis resistance in human tumors [30]. Our previous work suggested a link between RAB35 activity and increased breast cancer cell migration [22]. Although some studies showed that RAB35 has the opposite effect on the migration in some kind of cancer cells [17, 31], in the present work, we found that EGF induced RAB35 activation, while blocking RAB35 expression greatly abolished EGF-induced cell invasion. These results suggest that EGF might promote cell invasion in breast cancer cells by activating RAB35. Until now, limited knowledge was concerning the regulation of MICAL1 function by EGF signaling in breast cancer cells. In the current study, we determined that RAB35 and MICAL1 coimmunoprecipitated, and this interaction was disrupted when RAB35 was inactivated. We also observed that MICAL1 silencing delayed the increased invasive ability of RAB35 (CA)-expressing breast cancer cells. Moreover, knockdown RAB35 reduced ROS level as well as P-Akt level in breast cancer cells. Besides the fact that CC domain of MICAL-l1 interacts with active mutants of RAB35 [23], and the inhibitory effect of MICAL on ROS generation is thought to be dependent on the binding of CC domain to its LIM domain, therefore, we speculated that active form of RAB35 might be able to release MICAL1 auto-inhibition by directly binding to the CC domain of MICAL1, thereby allowing ROS generation and promoting cell migratory and invasive properties.

## Conclusions

Our results provide evidence that MICAL1 plays an essential role in the activation of ROS/Akt signaling and cell invasive phenotype and identify a novel link between RAB35 and MICAL1 in regulating breast cancer cell invasion. Although the current study has contributed to the mechanistic understanding the role of MICAL1 in breast cancer cell migration and invasion, the issue as to how RAB35 precisely regulates MICAL1 in breast cancer cells is unlikely to be settled in this paper. In conclusion, results obtained in this study clearly establish a new mechanistic connection between RAB35 and MICAL1 in the context of ROS generation, which could be essential in promoting cell migration and invasion during breast cancer cell metastasis.

## Abbreviations

CC, Coiled-coil; CH, Calponin homology; EGF, Epidermal growth factor; FAD, Flavin adenine dinucleotide; FBS, Fetal bovine serum; GAPDH, Glyceraldehyde 3-phosphate dehydrogenase; GAPs, GTPase activating proteins; GEFs, Guanine nucleotide exchange factors; LIM, Lin11, Isl-1 and Mec-3; MICAL1, Molecules interacting with CasL; qRT-PCR, Quantitative real time polymerase chain reaction; ROS, Reactive oxygen species; SDS-PAGE, Sodium dodecyl sulphate polyacrylamide gel electrophoresis
